# Neurologists’ Knowledge, Practice, and Attitudes towards Pharmacovigilance and Adverse Drug Reactions Reporting Process in Epileptic Patients—Comparative Analysis from Poland and Egypt

**DOI:** 10.3390/ijerph19074169

**Published:** 2022-03-31

**Authors:** Dorota Kopciuch, Nashwa Nabil Kamal, Nashaat Nabil Kamal, Nermin Aly Hamdy, Anna Paczkowska, Tomasz Zaprutko, Piotr Ratajczak, Jędrzej Fliciński, Krzysztof Kus, Elżbieta Nowakowska

**Affiliations:** 1 Department of Pharmacoeconomics and Social Pharmacy, Poznan University of Medical Sciences, 60-806 Poznan, Poland; aniapaczkowska@ump.edu.pl (A.P.); tomekzaprutko@ump.edu.pl (T.Z.); p_ratajczak@ump.edu.pl (P.R.); kkus@ump.edu.pl (K.K.); 2 Department of Public Health and Preventive Medicine, Faculty of Medicine, Minia University, Minia 61-511, Egypt; nashwa.farag@mu.edu.eg (N.N.K.); nashaat.kamal@must.edu.eg (N.N.K.); 3 Medical Biotechnology Department, College of Biotechnology, Misr University for Science and Technology (MUST), 6th of October City 12-573, Egypt; 4 Department of Neurology and Psychiatry, Minia University, Minya 61-511, Egypt; nermeen.ali@mu.edu.eg; 5 Department of Developmental Neurology, Poznan University of Medical Sciences, 60-355 Poznan, Poland; flicinski@hotmail.com; 6 Department of Pharmacology and Toxicology Institute of Health Sciences, Collegium Medicum, University of Zielona Gora, 65-417 Zielona Góra, Poland; elapharm@ump.edu.pl

**Keywords:** pharmacovigilance, epilepsy, adverse drug reactions, drug safety, neurologists’ knowledge, Poland, Egypt

## Abstract

Objectives: To compare neurologists’ knowledge, practice, and barriers of pharmacovigilance (PV) process among patients with epilepsy in Poland and Egypt. Methods: It was an international study that used an online questionnaire e-mailed to neurologists registered to practice in Poland and Egypt. Results: Most of the neurologists were familiar with the definition of PV and adverse drug reactions (ADRs), but relatively few neurologists knew where to report ADRs, especially the Egyptian neurologists. Only 31.11% of the neurologists from Egypt and 39.90% neurologists from Poland declared that they had reported ADRs at least once during their professional practice, and few of them declared the regular reporting of such incidents. The main reason for the neurologists not reporting ADRs was the lack of time and a conviction that reporting ADRs would be an additional burden that would generate extra work. Conclusion: The standards of pharmacovigilance process, safety control, and quality are not the same throughout the world. System-regulated PV stabilization in a country translates into the practice of maintaining PV. Monitoring the safety of pharmacotherapy and knowledge of risks associated with ADRs should be included in the academic curricula of physician courses.

## 1. Introduction

An adverse drug reaction (ADR) is defined by the World Health Organization (WHO) “as a response to a drug that is noxious and unintended and occurs at doses normally used in man for prophylaxis, diagnosis or therapy of disease, or modification of physiological function” [1]. An ADR has an unknown etiology, causing an enormous fiscal burden on both the society and healthcare system and contributes to around 5–20% of hospitalizations worldwide, so a well-established pharmacovigilance system plays a pivotal role in facing this situation [1,2,3,4]. The WHO has also indicated that epilepsy is one of the most frequent diseases among non-communicable diseases of the brain and affects more than 50 million people worldwide, 80% of whom reside in developing countries [5]. The mainstay of treatment in epilepsy is the use of antiepileptic drugs (AEDs) [6]. AEDs have different mechanisms of action and considerable interindividual pharmacokinetic variability and are susceptible to cause adverse effects and drug interactions, such as idiosyncratic reactions, dose-related neurocognitive effects and complications of long-term use [7,8].

Pharmacovigilance (PV) and the monitoring of ADRs help to evaluate the effectiveness and risk of medications, empower safe and rational use of drugs and enhance general patient care and well-being. Additionally, the cost of ADRs in the community is high, and underreporting of ADRs by health care professionals is a globally perceived issue [9,10,11]. The standards of pharmacovigilance process, safety control and quality are not the same or even similar throughout the world [12]. The highest and most rigorous standards are those in the most developed health systems in North America, Europe and Southeast Asia [12]. In many other countries, health, safety and quality regulations are still being introduced and/or fully implemented [13]. The establishment of pharmacovigilance systems in Arab countries has lagged behind that of developed countries. Eight out of fifteen countries launched their national PV programs in the past decade (2008–2018), showing that PV programs in Arab countries are still in their infancy stages, wherein stakeholder support and enforcement are needed [14]. Most of the requirements from guidelines of good pharmacovigilance practice (GVP) developed by the European Medicines Agency (EMA) and required in European countries have also been fulfilled by Arab countries. There are, however, a large part of the regulations that Arab authorities are still working on implementing, including guidelines for conducting PV among the pediatric population or during pregnancy [14].

Around 53% of the centers in Arab countries used reported data to change product information, to generate safety warnings, and to enact drug suspension or withdrawal, with Egypt being the country where most action was taken and that demonstrates the largest development potential in the PV context [15]. The number of reports submitted to the national PV centers in most countries has increased in recent years, especially in Egypt [16]. Egypt has been a member of the WHO International Program for Drug Monitoring since 2001, but although the Egyptian Pharmacovigilance Centre (EPVC) was established over 11 years ago, little is known about pharmacovigilance culture and practice among Egyptian health care practitioners (HCPs) [17].

Conversely, Poland seems to be a country with a well-founded position of PV in the country. Poland has been a member of the WHO International Program for Drug Monitoring since 1972, and the Office for Registration of Medicinal Products receives tens of thousands of ADRs reports annually [18].

It is assumed that, apart from the stabilized PV situation in a given health care system, effectively functioning PV depends on many other factors, such as cultural differences in attitudes towards ADR reporting, in particular the knowledge of and physicians’ attitude towards PV and ADRs [19].

In the case of epileptic patients, neurologists are the first to be informed about alarming symptoms by their patients, therefore the full participation and engagement of neurologists in the pharmacovigilance process in epileptic patients are crucial to ensure safe pharmacotherapy.

The aim of our study is to compare the neurologists’ knowledge, practice, and barriers of the pharmacovigilance process among patients with epilepsy in Poland and Egypt [19].

## 2. Methodology

This was an international study based on scientific collaboration between two Universities from Poland and Egypt. Due to the current COVID-19 pandemic and the resultant need to limit direct contact, the research tool used was an online anonymous questionnaire on Google Forms e-mailed to neurologists registered in the official database kept by the Supreme Chamber of Doctors from Poland and Egypt. A literature review was conducted before designing the questionnaire. Important questions and topics from the literature were either modified or directly included as items in our questionnaire. The questionnaire contains 19 items (nline questionnaire items covered the following: (1) characteristic of the study population, (2) knowledge of pharmacovigilance and adverse drug reactions (3) pharmacovigilance practice, (4) attitude to and (5) barriers to pharmacovigilance for patients with epilepsy, and also (6) activities to improve spontaneous ADR reporting.

The initial questionnaire was piloted in a sample of 20 neurologists across the country to assess face validity and clarity. The time needed to complete the questionnaire was recorded and all pilot study participants provided written feedback. The 5 min questionnaire was modified based on the pilot study results. Neurologists who did not complete the questionnaire within 3 weeks from the initial mailing were contacted a second time by e-mail. After the reminder, the questionnaire was e-mailed second time to any remaining non-responders.

The study was conducted between September 2021 and February 2022. Statistical analysis was performed using STATISTICA PL 10.0 (StatSOFT). The figures were expressed as the mean, SD, max, and min values. The data distribution pattern was not normal (unlike the Gaussian function). Significant differences between % of group results were determined by the analysis of Test for Proportions.

## 3. Results

The questionnaires were handed out to 2331 neurologists and only 1095 (656 from Poland and 439 from Egypt) expressed their consent to participate in the study by sending their responses. The response rate was 47%. The age of the neurologists ranged from 37 to 65 years (mean = 42.05 years). The duration of the neurologists’ practice ranged from 5 to 34 years (mean = 15.13 years). Most neurologists work at private practices and hospitals (Table 1). The average number of patients with epilepsy per day was 12.04 (Table 1).

### 3.1. Knowledge

Most of the neurologists were familiar with the definition of pharmacovigilance (PV) (PL—57.43%; EG—36.23%), the purpose of PV (PL—64.02%; EG-45.12%), its functions (PL—72%; EG—32.92%) and also the definition of ADRs (PL—43.76%; EG—65.02%) and the purpose of ADRs (PL—46.12%; EG—55.09%) (Table 2). Knowledge of the above issues was correlated with age (*p* < 0.05), years of professional experience (*p* ˂ 0.05) and place of employment (*p* ˂ 0.05), i.e., younger neurologists with shorter professional experience and those who work at universities had a better knowledge of the above-mentioned definitions (Table 2); however, it should be noted that older Egyptian neurologists presented better knowledge of the subject than Polish neurologists and, interestingly, it was not related to the length of their experience (Table 2). Additionally, an interesting observation was that the best knowledge was demonstrated by neurologists working at universities in comparison to neurologists working in other places admitted in the study (Table 2).

Most of the respondents knew when (PL—48.98%; EG—44.34%) to report adverse reactions and felt co-responsible for reporting them (PL—63.34%; EG—48.09%), but relatively few neurologists knew where to report ADRs, especially among the Egyptian neurologists (Table 2). These results were correlated with sociodemographic data (*p* < 0.05) (Table 2).

### 3.2. Attitude

Only 32.98% neurologists from Poland, and 42.34% from Egypt believe that many ADRs are preventable, but most of the neurologists (PL—77.09%; EG—70.09%) believe it is necessary to report ADRs from patients with epilepsy and the ADR reporting is a neurologist’s obligation (PL—70.09%; EG—60.13%) (Table 2).

### 3.3. Practice

Only 31.11% of the neurologists from Egypt and 39.90% neurologists from Poland declared that they had reported ADRs at least once during their professional practice, and few of them (PL—32.78%; EG—28.89%) declared the regular reporting of such incidents. Most of the neurologists reported only ˂5 ADRs in the last 6 months (PL—82.98%; EG—76.12%), mostly severe (PL—73.34%; EG—65.09%) and rare (PL—13.65%; EG—25.34%) ADRs. It was correlated with sociodemographic data (*p* < 0.05), especially with age and years of experience (Table 2).

### 3.4. Communication Method Preferred by Neurologists to Send ADRs

**Figure 1:**
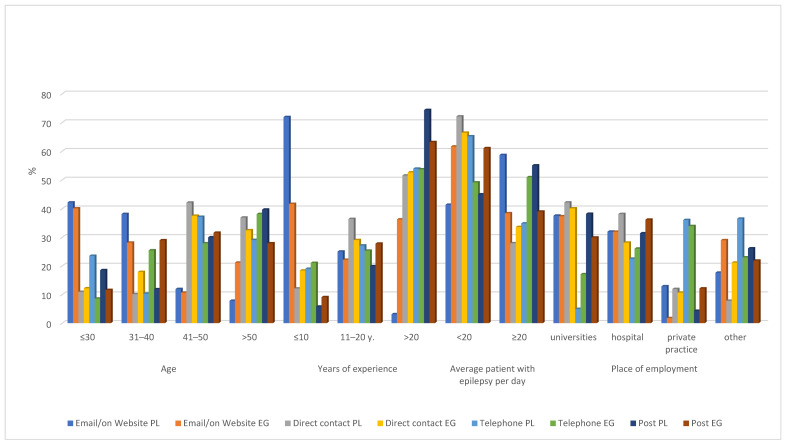
The most popular communication method preferred by neurologists to send adverse drug reactions (ADRs) to an ADR reporting center.

The most popular communication methods preferred by neurologists to send ADRs to an ADR reporting center were e-mail or website (PL—47.09%; EG—37.12%), especially by the youngest neurologists with shorter years of experience. On the other hand, the oldest neurologists preferred the traditional mail service as an option to report ADRs (Figure 1 and Supplementary Materials).

### 3.5. Sources Used to Gather Information about ADRs

**Figure 2:**
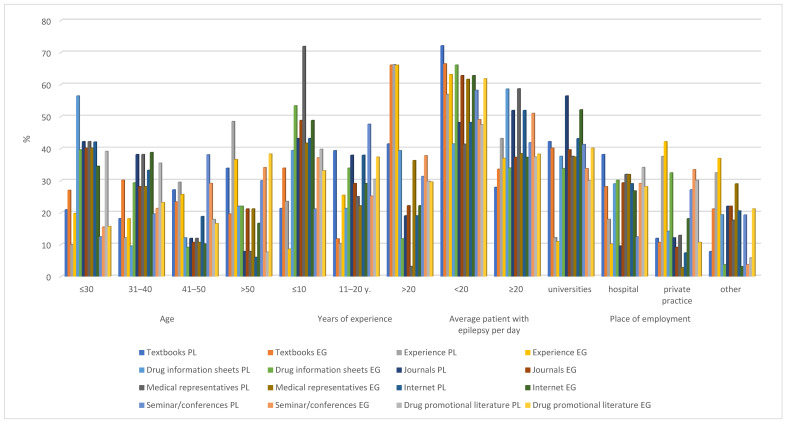
The main sources used to gather information about adverse drug reactions.

The main sources used to gather information about ADRs by the Polish neurologists included the Internet (78.09%), experience (37.02%) and seminars/conferences (31.12%). Among the Egyptian neurologists, the most popular sources of information about ADRs were also the Internet (82.12%), seminars/conferences (37.32%), and drug information sheets (30.12%) (Figure 2 and Supplementary Materials).

### 3.6. The Main Factors Discouraging Neurologists from Delivering Pharmacovigilance

The main reason for the neurologists not to report ADRs was the lack of time for such activities (PL—37.09%; EG—35.12%) and a conviction that reporting ADRs would be an additional burden generating extra work (PL—23.09%; EG—25.12%). It was correlated with sociodemographic data (*p* < 0.05) (Table 3).

The largest percent of neurologists claim that the institutional role should be more active to improve the PV system in practice. It was declared by 36.65% of the Polish neurologists and 30.45% neurologists from Egypt. Other activities that should be implemented were compulsory in-service ADR reporting trainings (Table 3). A total of 21% of the neurologists from Poland and 19.54% from Egypt also indicated “Strengthen training program on ADR reporting” as other activities that should be implemented into health systems to improve PV (Table 3).

## 4. Discussion

In the authors’ opinion, the results of this study are innovative because, after the review of the available scientific literature within this scope, it was found that no analysis has been performed to date to address the pharmacovigilance process among neurologists directly. Moreover, the innovative nature of this study has been strengthened by the comparison of the results of the analysis conducted in two countries. The selection of Poland and Egypt for the analysis was justified by an attempt to assess whether the effect of the “well-grounded position of PV” in the existing healthcare system affects the observance of the obligation to participate in the national PV and ADR reporting by neurologists.

Due to the lack of literature where the study group would only be composed of neurologists, the discussion was referenced to the literature that addressed the analogous research problem; however, the study was conducted without a division of the study group by specialization and referred to the general professional group, i.e., medical doctors.

The results of the study show that the neurologists had a good knowledge of the definition and the purpose of PV. It was similar to observations made by Hardeep et al. [20] and Lakshman Das et al. [21], who have shown that physicians, in general, have a good knowledge of medicinal safety, ADRs, and the obligation of the PV process. The results also show that the neurologists from our study had an idea about medication safety and ADR reporting, both from Poland and Egypt, and such knowledge was dependent on the age of the respondents. This is very similar to a study in northern Sweden, by Backstrom in 2000 [22]. Despite such a good knowledge of PV and ADRs, the neurologists from our study were not aware of the pharmacovigilance centers responsible for PV in their countries [23]. It was especially observed among the neurologists from Egypt. These findings are consistent with a study in Malaysia, where it was the main predictor of underreporting of ADRs by physicians. In this study, about 40% of the respondents were completely unaware of national pharmacovigilance centers; hence, they did not report ADRs [24,25,26]. This shows the lack of communication between the administrative bodies of the centers and the medical staff. One of the measures to address this issue is to introduce PV as an essential part of the training of healthcare professionals, especially among physicians. Furthermore, national PV centers should publicize their activities among physicians, as has been mentioned in the literature [27]. The vast majority of the neurologists, especially from Egypt, did not acknowledge the contribution of other health care professionals as potential ADR reporters [28,29], but they knew about their responsibility of including PV into their daily duties.

Additionally, most of the neurologists knew about the time frame of serious ADR reporting, but it was not correlated with the number of reported ADRs observed in patients with epilepsy because a great majority declared that during the last 6 months they had reported only ˂5 ADR. This may be caused by numerous systemic factors, indicated by both the Polish and Egyptian neurologists as barriers in PV processing. Among the barriers in conducting PV, the neurologists participating in our study most frequently named the lack of time to fill in a report or a concern that the report would generate extra work. This was similar to other studies. Various studies [30,31] found that the main reason for underreporting ADRs was the clinical negligibility of the adverse reaction, lack of time, and little knowledge of the types of reactions to be preferentially reported. In our study, the vast majority of neurologists also reported the absence of a fee for reporting ADRs as the main barriers of PV providing, similar to the study by Venugopal [28].

The findings from the study suggest a positive attitude of neurologists towards reporting an ADR, which is very positive indeed, as was observed in other studies, where participants were willing to learn and apply ADR reporting knowledge in their work setting [32]. It was similar among the neurologists from Poland and Egypt, as well. While the majority of doctors from Poland felt that ADR reporting was a professional obligation, 43.32% of neurologists from Egypt disagreed. This may be caused by an unstable PV situation in Egypt. Additionally, a large percentage of neurologists, especially from Egypt, believed that only serious ADRs should be considered more important or they did not even know what type of ADR to report. This was similar to previous studies [33,34]. It is important to acknowledge that less serious and unusual ADRs are also important because they might serve as a clue to the possibility of a fatal ADR occurring in the future. The factors identified by doctors as obstacles in reporting ADRs should be dealt with immediately, they included the previously mentioned barriers [28].

We also asked about the regularity of ADRs reporting and the number of ADRs reported in 6 months and the most frequent response from the neurologists was “yes”, but the most frequent reply to the second question was ˂5. According to various research outcomes, doctors’ practice towards ADR reporting was far below expectations. Meanwhile, the rate at which ADRs were reported to the relevant regulatory authority was quite overwhelming; a greater part of the doctors that came across ADR either sent few reports or did not report at all [35]. Surveys performed in Malaysia have shown that only 5.3% of doctors had ever reported ADRs [35]; a similar result was found in UAE 11% [36] and Romania 15% [37].

Similarly, a survey from Romania revealed that 79.9% of the interviewed doctors did not report any ADR [38], and a comparable result was obtained in India at 77% [39]. In contrast, an article from Sweden has a positive finding, with 62% having ever reported an ADR [33].

The study results indicate the sources most frequently used to gather information about ADRs. The most popular source of information used by the neurologists from Poland was the Internet and experience, and seminars, and from Egypt, it was journals and also the Internet. Surveys carried out in Pakistan have shown that 24% of doctors refer to the Internet, 33.6% to seminars, 18.4% to journals, and 10.4% to drug adverts [25]. Similarly, in Nigeria, 41.4% refer to books/journals, 18.3 to seminars/training, 4.4% to the Internet [40], and in India, 63% of doctors identified the Internet as the source of information, 65% seminars, 69% journals, 40% medical books [39], and other doctors (89%) emphasized the role of information technology [34], at 93.6% [41], and 75% [42].

In this study, it was observed that there are differences in the maturity of the systems and in the activities conducted in the programs. One of the possible reasons is the availability of qualified trained personnel as seen in Egypt, and in Poland.

The neurologists in our study suggested different activities that should be implemented for the improvement of the ADR reporting system, including strengthening the training program on ADR reporting, activating institutional role in ADR reporting, and also including reporting exercises in the undergraduate examination as an important tool for increasing physicians’ awareness of ADRs in practice. In 2009, Oshikoya and Awobusuyi also recommended including pharmacovigilance as a topic in continuing education programs [29]. Various studies have shown that the optimization of the knowledge, attitude, and practices about pharmacovigilance is essential to promote reporting [43,44].

The participants also urged the governments to take vital steps to ensure safe and effective medicine utilization among the population. Furthermore, the neurologists also suggested reforms for the improvement of the ADR reporting system, including continuous education, seminars, as well as training courses. The literature also supports that a provision of optimal knowledge, awareness of attitude, and practices related to pharmacovigilance are essential to promote ADR reporting [43,44]. Globally, there is a shift in ADR reporting from the prescriber to the consumer or patient in developed countries. However, in developing countries, such as Egypt, the ADR reporting system is still at its infancy stage [45,46].

Eight out of fifteen Arab countries launched their national PV programs in the past decade (2008–2018), showing that PV programs in Arab countries are still in their infancy stages, wherein stakeholder support and enforcement are needed [14].

## 5. Conclusions

To conclude, monitoring the safety of pharmacotherapy and knowledge of risks associated with ADRs should be included in the academic curricula of physician courses. Neurologists from Poland and Egypt have good knowledge of pharmacovigilance or ADR reporting. Both the Polish and Egyptian neurologists demonstrated a positive attitude towards ADR reporting and understood the importance of PV in the general concept of ensuring pharmacotherapy safety to patients with epilepsy. However, it seems that system-regulated PV stabilization in a country translates into the practice of conducting PV because it has been observed that, despite the high level of knowledge, and neurologists’ positive attitude towards PV in Poland and in Egypt, the Polish neurologists had better grounded practice in conducting PV.

## Figures and Tables

**Table 1:** Demographic characteristics of neurologists (*n* = 1095).

Parameter	PL/EG	General
Age [years; mean (SD)]	45.09 (30.01)/ 56 (33.12)	42.05 (38.32)
Sex [female; N (%)]	374 (57)/ 167 (38)	752 (68.67)
Years of practice [years; mean (SD)]	17.87 (10.01/ 15.98 (12.12)	15.13 (11.12)
Place of employment; N (%)		
Universities	104 (16.01)/ 57 (13.77)	118 (10.77)
Hospital	197 (30.12)/ 123 (28.14)	248 (22.64)
Private practice/private office	288 (44.23)/ 162 (37.01)	575 (52.51)
Other	67 (10.12)/ 97 (22.05)	154 (14.06)
Patient with epilepsy per day [mean (SD)]	15.02 (6.34)/ 10.88 (7.07)	12.04 (8.12)

**Table 2:** Assessment of neurologists’ knowledge about pharmacovigilance and adverse drug reactions (*n* = 1095).

	Response N (%) PL/EG	Country/ *p*-Value	Age (%)				Years of Experience (%)			Average Patient with Epilepsy Per Day (%)		Place of Employment (%)			
	656 (60)/ 439 (40)		≤30	31–40	41–50	>50	≤10	11–20	>20	<20	≥20	Universities	Hospital	Private Practice/Private Office	Other
Knowledge															
The most appropriate definition of pharmacovigilance: Correct answer	377 (57.43)/ 159 (36.23)	PL	42.01 *	33.23	18.78	5.98	43.12 ^c^	37.89	18.99	48.12	51.88	43.09 ^	29.03	7.37	20.51
		EG	34.44 ^a^	38.76	10.16	16.64	48.76 ^c^	29.09	22.15	62.77 ^f^	37.23	52.08 #	26.77	18.09	3.06
		*p*-value:	NS	NS	0.0134	0.0001	NS	0.0515	NS	0.0019	0.0019	NS	NS	0.0002	<0.0001
The most appropriate purpose of pharmacovigilance: correct answer	420 (64.02)/ 198 (45.12)	PL	29.97 *	34.12	30.10	5.81	41.76	28.44	29.80	58.09	41.91	37.12 ^	29.04	15.12	18.72
		EG	40.09 ^a^	28.12	10.65	21.14	33.10	37.32	29.58	55.90	44.10	27.12	31.87 #	33.76	7.25
		*p*-value	0.0127	NS	<0.0001	<0.0001	0.0394	0.0263	NS	NS	NS	0.0143	NS	<0.0001	0.0002
Functions of pharmacovigilance: correct answer	472 (72.00)/ 145 (32.92)	PL	44.09 *	25.12	28.10	2.69	36.87	41.10 ^c^	22.03	53.50	46.50	39.21 ^	27.03	8.66	25.10
		EG	29.10	37.31	18.87	14.72	44.09 ^c^	29.68	26.23	65.90 ^f^	34.10	47.14 ^	26.05	5.72	21.09
		*p*-value	0.0013	0.0042	0.0265	<0.0001	NS	0.0134	NS	0.0084	0.0084	NS	NS	NS	NS
Definition of ADR: correct answer	287 (43.76) 285/ (65.02)	PL	39.10 *	35.43	17.85	7.62	39.76	30.44	29.80	47.43	37.34	29.97	34.12 #	30.10	5.81
		EG	22.65	29.13	19.95	28.27	33.10	37.32	29.58	61.77	38.23	40.09 ^	28.12	10.65	21.14
		*p*-value	<0.0001	NS	NS	<0.0001	NS	NS	NS	0.0006	NS	0.0112	NS	<0.0001	<0.0001
The purpose of an ADR: correct answer	303 (46.12)/ 242 (55.09)	PL	37.55 ^a^	31.96	12.87	17.62	38.46	36.12	25.42	28.99 ^f^	71.01	30.97	34.12 ^	31.10	3.81
		EG	40.34 *	31.92	17.76	9.98	48.87 ^c^	33.32	17.81	43.71	56.29	40.09 ^	28.12	10.65	21.14
		*p*-value	NS	NS	NS	0.0112	0.0148	NS	0.0332	0.0004	0.0004	0.0266	NS	<0.0001	<0.0001
When serious ADRs should be reported?: Correct answer	321 (48.98)/ 195 (44.34)	PL	43.30 *	28.98	19.10	7.62	41.38 ^c^	21.30	37.32	41.43	58.57	56.41 &	9.52	12.09	21.98
		EG	29.90	35.12 ^a^	12.45	22.53	56.34 ^c^	31.90	11.76	66.06 ^f^	33.94	39.59 ^	29.31	9.12	21.98
		*p*-value	0.0024	NS	0.0489	<0.0001	0.0010	0.0072	<0.0001	<0.0001	<0.0001	0.0002	<0.0001	NS	NS
To whom should ADRs be reported?: Correct answer	256 (39.10)/ 104 (23.77)	PL	38.46 *	26.12	33.65	1.77	41.90 ^c^	39.36	18.74	41.18	58.82	37.55	31.96	12.87	17.62
		EG	28.87	33.32 *	28.17	9.64	31.10	39.09	29.81	52.57	47.43	37.34 ^	31.92	1.76	28.98
		*p*-value	NS	NS	NS	0.0006	NS	NS	0.0214	0.0487	0.0487	NS	NS	0.0012	0.0163
Are you co-responsible for ADR reporting?: Yes	415 (63.34)/ 211 (48.09)	PL	33.76 *	29.12	33.43	3.69	44.10 ^c^	38.12	17.78	28.98 ^f^	71.02	29.41	42.88 ^	9.62	18.09
		EG	41.23 *	12.38	27.15	19.24	29.09	31.65	39.26	36.47 ^f^	63.53	37.33	26.15	15.43	21.09
		*p*-value	NS	<0.0001	NS	<0.0001	0.0035	NS	<0.0001	NS	NS	0.0448	<0.0001	0.0317	NS
Attitude															
Do you believe that many ADRs are preventable?: yes	216 (32.98)/ 186 (42.34)	PL	33.34	38.51 ^a^	12.54	15.61	40.04	26.61	33.35	44.09	55.91	39.90 ^#	35.12	12.45	12.53
		EG	22.57	26.12	33.74	17.57	33.98	31.61	34.41	56.32	43.68	12.05 #&	29.76	12.76 ^&	63.24
		*p*-value	0.0169	0.0083	<0.0001	NS	NS	NS	NS	0.0145	0.0145	<0.0001	NS	NS	<0.0001
Do you think it is necessary to report ADRs from patients with epilepsy?: yes	506 (77.09)/ 308 (70.09)	PL	43.30 *	28.98	19.10	7.62	41.38	21.30	37.32	41.43	58.57	56.41 & ^	9.52	12.09	21.98
		EG	39.90 *	35.12	12.45	12.53	53.34	33.90	11.76	66.06 ^f^	33.94	39.59 ^	29.31	9.12	21.98
		*p*-value	NS	NS	0.0134	0.0204	0.0009	0.0001	<0.0001	<0.0001	<0.0001	<0.0001	<0.0001	NS	NS
Do you think the ADR reporting is a neurologist’s obligation?: yes	460 (70.09)/ 264 (60.13)	PL	4.01	33.23 ^g^	18.78	7.98	43.12	37.89	18.99	48.12	51.88	43.09 ^	29.03	6.37	21.51
		EG	34.44	38.76 ^a^	10.16	16.64	48.76	29.09	22.15	62.77 ^f^	37.23	52.08 #	26.77	18.09	3.06
		*p*-value	<0.0001	NS	0.0021	0.0004	NS	0.0166	NS	0.0003	0.0001	0.0196	NS	<0.0001	<0.0001
Practice															
Have you ever reported any ADRs from patient with epilepsy?: yes	262 (39.90)/ 137 (31.11)	PL	39.40 *	31.23	18.78	10.59	38.12	43.89 ^c^	17.99	41.12	58.88	43.09 ^	29.03	7.37	20.51
		EG	44.14 *	28.76	17.76	9.34	43.76 ^c^	32.69	23.55	60.77 ^f^	39.23	52.08 #	26.77	18.09	3.06
		*p*-value	NS	NS	NS	NS	NS	0.0302	NS	0.0002	0.0002	NS	NS	0.0012	<0.0001
Do you report ADRs on a regular basis from patients with epilepsy?: yes	215 (32.78)/ 127 (28.89)	PL	42.15 *	38.12	11.92	7.81	31.76	38.44	29.80	58.09	41.91	37.12 ^	29.04	15.12	18.72
		EG	40.09 ^a^	28.12	10.65	21.14	33.10	37.32	29.58	55.90	44.10	27.12 #	31.87	33.76	7.25
		*p*-value	NS	NS	NS	0.0004	NS	NS	NS	NS	NS	NS	NS	0.0001	0.0036
If yes, how many ADRs on average would be diagnosed (or observed) in a period of 6 months?															
<5	544 (82.98)/ 334 (76.12)	PL	29.10	37.31 *	18.87	14.72	44.09	29.68	26.23	65.90 ^f^	34.10	47.14 ^	26.05	5.72	21.09
		EG	44.09 *	25.12	28.10	2.69	36.87	41.10 ^f^	22.03	53.50	46.50	39.21 ^	27.03	8.66	25.10
		*p*-value	<0.0001	0.0002	0.0014	<0.0001	0.0349	0.0005	NS	0.0003	0.0003	0.0216	NS	NS	NS
5–10	79 (12.01)/ 75 (17.12)	PL	56.41 ^h^	9.52	12.09	21.98	51.11 ^d^	17.58	31.31	58.17	41.83	41.23 &	12.38	27.15	19.24
		EG	39.59 ^a^	29.31	9.12	21.98	37.10	25.15	37.75	49.03	50.97	33.76 #	29.12	33.43	3.69
		*p*-value	0.0368	0.0018	NS	NS	NS	NS	NS	NS	NS	NS	0.0102	NS	0.0027
>10	33 (5.01)/ 30 (6.76)	PL	39.10 *	35.43	17.85	7.62	39.76	30.44	29.80	47.43	37.34	29.97 #	34.12	30.10	5.81
		EG	22.65	29.13	19.95	28.27	33.10	37.32	29.58	61.77 ^f^	38.23	40.09 ^	28.12	10.65	21.14
		*p*-value	NS	NS	NS	0.0310	NS	NS	NS	NS	NS	NS	NS	NS	NS
What type of ADR is the one you report most frequently?															
Severe	481 (73.34)/ 286 (65.09)	PL	37.55 *	31.96	12.87	17.62	39.38	21.30	39.32	41.43	58.57	37.55 ^	29.00	14.14	19.31
		EG	37.34 ^a^	31.92	1.76	28.98	53.34 ^c^	33.90	11.76	66.06 ^f^	33.94	33.76 #	30.12 #	32.43 #	3.69
		*p*-value	NS	NS	<0.0001	0.0002	0.0002	0.0001	<0.0001	<0.0001	<0.0001	NS	NS	<0.0001	<0.0001
Rare	89 (13.65)/ 111 (25.34)	PL	6.64 *	20.10	28.12	45.14	43.12 ^c^	37.89	18.99	48.12	51.88	56.41 &	9.52	12.09	21.98
		EG	3.54 *	28.90	31.90	35.66	48.76 ^c^	29.09	22.15	62.77 ^f^	37.23	39.59 ^	29.31	9.12	21.98
		*p*-value	NS	NS	NS	NS	NS	NS	NS	NS	NS	NS	NS	NS	NS
Unexpected	85 (13.01)/ 42 (9.57)	PL	20.89	18.12	27.12	33.87	29.56	31.17	39.27	51.09	48.91	37.55 ^	31.96 ^	12.87	17.62
		EG	26.98	30.13	23.34	19.56	33.13	39.03	27.84	68.02 ^f^	31.98	37.34 ^	31.92 ^	1.76	28.98 ^
		*p*-value	NS	NS	NS	NS	NS	NS	NS	NS	NS	NS	NS	0.0415	NS

PL—Poland; EG—Egypt; NS—not statistically significant difference (*p* > 0.05); * statistically significant difference (*p* < 0.05) vs. >50 y.o.a; ^c^ statistically significant difference (*p* < 0.05) vs. >20 years; ^ statistically significant difference (*p* < 0.05) vs. private practice/private office; ^a^ statistically significant difference (*p* < 0.05) vs. 41–50 y.o.a; ^d^ statistically significant difference (*p* < 0.05) vs. 11–20 years; ^f^ statistically significant difference (*p* < 0.05) vs. ≥20 average patient/day; ^g^ statistically significant difference (*p* < 0.05) vs. ≤30 y.o.a.; ^h^ statistically significant difference (*p* < 0.05) vs. 31–40 years; # statistically significant difference (*p* < 0.05) vs. other; & statistically significant difference (*p* < 0.05) vs. hospital.

**Table 3:** The factors that may discourage neurologists from delivering pharmacovigilance and activities to improve spontaneous ADR reporting (*n* = 1095).

	Response N(%) PL/EG	Country	Age (%) PL/EG				Years of Experience (%) PL/EG			Average Patient with Epilepsy Per Day (%) PL/EG		Place of Employment (%) PL/EG			
			≤30	31–40	41–50	>50	≤10	11–20	>20	<20	≥20	Universities	Hospital	Private Practice/Private Office	Other
Barriers															
Apprehension about sending an inappropriate report	40 (6.09)/ 25 (5.81)	PL	35.77 ^a^	29.34	15.94	18.95	35.55	38.05	26.40	34.98 ^f^	65.02	36.66 #^	37.94	15.09	10.31
		EG	28.90	31.03 *	28.58	11.49	30.00	38.19	31.81	61.55 ^f^	38.45	33.76 #	29.12 #	33.43 #	3.69
		*p*-value	NS	NS	NS	NS	NS	NS	NS	0.0363	0.0363	NS	NS	NS	NS
Lack of time to fill in a report	243 (37.09)/ 154 (35.12)	PL	12.41	19.57	38.04 ^g^	29.98	21.11	47.58 ^@^	31.31	58.17	41.83	41.23 &	12.38	27.15	19.24
		EG	15.49	21.31	29.12 ^g^	34.08	37.10	25.15	37.75	49.03	50.97	33.76 #	29.12 #	33.43 #	3.69
		*p*-value	NS	NS	NS	NS	0.0005	<0.0001	NS	NS	NS	NS	<0.0001	NS	<0.0001
Concern that the report will generate extra work	151 (23.09)/ 110 (25.12)	PL	39.10 *	35.43	17.85	7.62	39.76	30.44	29.80	47.43	37.34	29.97 #	34.12 #	30.10 #	5.81
		EG	15.65	23.13	22.95	38.27 ^g^	33.10	37.32	29.58	61.77 ^f^	38.23	40.09 ^	28.12	10.65	21.14
		*p*-value	<0.0001	0.0327	NS	<0.0001	NS	NS	NS	0.0218	NS	NS	NS	0.0002	0.0002
Absence of a fee for reporting ADRs	79 (12.01)/ 66 (15.09)	PL	46.09 *	34.20	11.90	7.81	71.88 **	25.00	3.12	41.35	58.65	37.55 ^	31.96	12.87	17.62
		EG	40.09 ^a^	28.12	10.65	21.14	41.65 ^d^	22.12	36.23	61.61 ^f^	38.39	37.34 ^	31.92 ^	1.76	28.98 ^
		*p*-value	NS	NS	NS	0.0208	0.0002	NS	<0.0001	0.0151	0.0151	NS	NS	0.0131	NS
Level of knowledge makes it difficult to decide whether an ADR has occurred	54 (8.25)/ 39 (8.98)	PL	38.09 *	37.15	18.78	5.98	43.12 **	37.89	18.99	38.00 ^f^	62.00	43.09 ^	29.03 ^	7.37	20.51 ^
		EG	38.09 ^a^	32.27	10.00	19.64	48.76 **	29.09	22.15	62.77 ^f^	37.23	52.08 # ^	26.77 #	18.09 #	3.06
		*p*-value	NS	NS	NS	0.0432	NS	NS	NS	0.0183	0.0183	NS	NS	NS	0.0141
Do not feel the need to report reactions reported by patients	36 (5.55)/14 (3.09)	PL	12.41	19.57	38.04 ^g^	29.98	21.11 **	31.31	47.58	58.17	41.83	27.15	12.38	41.23 &	19.24
		EG	15.49	21.31	29.12	34.08 ^g^	37.10	25.15	37.75	49.03	50.97	3.69	29.12 $	33.43 $	33.76 $
		*p*-value	NS	NS	NS	NS	NS	NS	NS	NS	NS	NS	NS	NS	NS
Physicians’ yellow cards not available when needed	52 (7.92)/30 (6.79)	PL	42.01 *	33.23	18.78	5.98	43.12 **	37.89	18.99	48.12	51.88	43.09 ^	29.03 ^	7.37	20.51 ^
		EG	34.44	38.76	10.16	16.64	48.76 **	29.09	22.15	62.77 ^f^	37.23	52.08 #	26.77 #	18.09 #	3.06
		*p*-value	NS	NS	NS	NS	NS	NS	NS	NS	NS	NS	NS	NS	0.0289
Activities															
Strengthen training program on ADR reporting	138 (21.07)/ 86 (19.54)	PL	39.10 ^a^	25.43	15.35	20.12	39.76	30.44	29.80	47.43	37.34	29.97 #	34.12 #	30.10 #	5.81
		EG	13.62	23.13	23.98	39.27 ^g^	33.10	37.32	29.58	61.77 ^f^	38.23	40.09 ^	28.12 ^	10.65	21.14
		*p*-value	<0.0001	NS	NS	0.0018	NS	NS	NS	0.0365	NS	NS	NS	0.0007	0.0005
ADR reporting should be compulsory in-service training	157 (23.96)/ 93 (21.27)	PL	36.41 ^a^	29.52	12.09	21.98	39.38	21.30	39.32	41.43	58.57	33.55	25.00	22.14	19.31
		EG	39.59 ^a^	29.31	9.12	21.98	53.34 **	33.90	11.76	66.06 ^f^	33.94	33.76 #	30.12 #	32.43 #	3.69
		*p*-value	NS	NS	NS	NS	0.0318	0.0261	<0.0001	0.0002	0.0002	NS	NS	NS	0.0005
Institutional role should be more active	240 (36.65)/ 134 (30.45)	PL	14.77	15.88	51.45 ^g^	17.09	10.87 **	32.15	56.98	73.98 ^f^	26.02	44.98 ^	31.09 ^	3.49	20.44 ^
		EG	14.50	13.46	29.04	43.00 ^g^	12.79 **	39.90	47.31	57.22	42.78	38.86 ^	29.49	13.60	18.05
		*p*-value	NS	NS	<0.0001	<0.0001	NS	NS	NS	0.0009	0.0009	NS	NS	0.0003	0.5765
Report forms should be included in prescribing pad	45 (6.88)/ 54 (12.31)	PL	9.93	12.12	29.51	48.44 ^g^	23.51	10.30	66.19 ^d^	56.88	43.12	12.17	17.88	37.51 $	32.44
		EG	19.74	18.06	25.66	36.54 ^h^	8.57 **	25.43	66.00	63.09	36.91	10.90	10.12	42.10 $ &	36.88 & $
		*p*-value	NS	NS	NS	NS	0.0401	0.0538	NS	NS	NS	NS	NS	NS	NS
An uncomplicated reporting system with quick feedback	25 (3.74)/ 47 (10.81)	PL	50.41 ^h^	8.52	19.09	21.98	39.38 ^d^	21.30	39.32 ^d^	41.43	58.57	32.55	33.00	15.15	19.31
		EG	34.59 ^j^	25.31	18.12	21.98	53.34 **	33.90	11.76	66.06 ^f^	33.94	33.76 #	30.12 #	32.43 #	3.69
		*p*-value	NS	NS	NS	NS	NS	NS	0.0066	0.0441	0.0441	NS	NS	NS	0.0283
Exercises should be included in undergraduate examination	51 (7.70)/ 25 (5.62)	PL	16.90	2.12	45.10 ^h^	35.88 ^h^	12.10 **	36.39	51.51	61.09 ^f^	38.91	42.15 #	38.12 #	11.92	7.81
		EG	13.17	10.88	43.51 ^h^	32.44 ^h^	18.37 **	29.03	52.60	66.43 ^f^	33.57	40.09 ^	28.12 ^	10.65	21.14
		*p*-value	NS	NS	NS	NS	NS	NS	NS	NS	NS	NS	NS	NS	NS

PL—Poland; EG—Egypt; NS—not statistically significant difference (*p* > 0.05); * statistically significant difference (*p* < 0.05) vs. >50 y.o.a; ^ statistically significant difference (*p* < 0.05) vs. private practice/private office; ^a^ statistically significant difference (*p* < 0.05) vs. 41–50 y.o.a; ^d^ statistically significant difference (*p* < 0.05) vs. 11–20 years; ^f^ statistically significant difference (*p* < 0.05) vs. ≥20 average patient/day; ^g^ statistically significant difference (*p* < 0.05) vs. ≤30 y.o.a.; ^h^ statistically significant difference (*p* < 0.05) vs. 31–40 y.o.a.; ^j^ statistically significant difference (*p* < 0.05) vs. 41–50 y.o.a; # statistically significant difference (*p* < 0.05) vs. other; & statistically significant difference (*p* < 0.05) vs. hospital; $ statistically significant difference (*p* < 0.05) vs. universities; ^@^ statistically significant difference (*p* < 0.05) vs. ≤10 years; ** statistically significant difference (*p* < 0.05) vs. >20 years.

## Data Availability

Not applicable.

## Supplementary Materials

The following are available online at https://www.mdpi.com/article/10.3390/ijerph19074169/s1, Table S1: The most popular communication method preferred by neurologists to sent ADRs to an ADRs reporting center.
