# Supplementary material for: Neurologists’ Knowledge, Practice, and Attitudes towards Pharmacovigilance and Adverse Drug Reactions Reporting Process in Epileptic Patients—Comparative Analysis from Poland and Egypt
Source: Int J Environ Res Public Health. 2022 Mar 31;19(7):4169. doi: 10.3390/ijerph19074169 (PMC8998900; doi:10.3390/ijerph19074169)
Supplement: Supplementary file 1 [file ijerph-19-04169-s001.zip › ijerph-1602434-supplementary.pdf]

Table S1. The most popular communication method preferred by neurologists to sent ADRs to an ADRs reporting center.

	Response N (%) PL/EG	Country/ *p*-Value	Age (%)				Years of experience (%)			Average Patient with Epilepsy per Day (%)		Place of Employment (%)			
	656 (60)/ 439 (40)		≤30	31–40	41–50	>50	≤10	11–20	>20	<20	≥20	Universities	Hospi-tal	Private Practice/ Private Office	Other
**Which method would you prefer to send ADRs information to an ADR reporting center?**															
Email/on Website	309(47.09)/ 163(37.12)	PL	42.15 *	38.12	11.92	7.81	71.88 [c]	25.00	3.12	41.35	58.65	37.55 ^	31.96 ^	12.87	17.62
		EG	40.09 *	28.12	10.65	21.14	41.65	22.12	36.23	61.61 [f]	38.39	37.34 ^	31.92 ^	1.76	28.98 ^
		p-value	NS	0.0300	NS	<0.0001	<0.0001	NS	<0.0001	<0.0001	<0.0001	NS	NS	0.0001	0.0043
Direct contact	119(18.23)/ 106(24.21)	PL	10.90	10.12 [j]	42.10	36.88	12.10 [c]	36.39	51.51	72.09 [f]	27.91	42.15 #	38.12 #	11.92	7.81
		EG	12.17 [a]	17.88	37.51	32.44	18.37 [c]	29.03	52.60	66.43 [f]	33.57	40.09 ^	28.12	10.65	21.14
		p-value	NS	NS	NS	NS	NS	NS	NS	NS	NS	NS	NS	NS	0.0041
Telephone	165(25.12)/ 132(30.01)	PL	23.51	10.30 [j]	37.13	29.06	18.98	27.13	53.89	65.17 [f]	34.83	4.96	22.51	36.03 $	36.50 $
		EG	8.57 *	25.43	27.88	38.12	21.03 [c]	25.31	53.66	49.09	50.91	17.09	26.04	33.91	22.96
		p-value	0.0006	0.0006	NS	NS	NS	NS	NS	0.0053	0.0053	0.0006	NS	NS	0.0118
Post	63(9.56)/ 42(9.66)	PL	18.51	11.84 *	29.98	39.67	5.76 [c]	19.90	74.34	44.98	55.02	38.19 ^	31.39	4.30	26.12
		EG	11.58 [a]	28.95	31.59	27.88	9.08 [c]	27.77	63.15	61.04	38.96	29.90	36.15 ^	12.12	21.83
		p-value	NS	0.0042	NS	0.0549	NS	NS	NS	0.0155	0.0155	NS	NS	NS	NS
**The sources used to gather information about ADRs:**															
textbooks	66(10.10)/ 73(16.55)	PL	20.89	18.12	27.12	33.87	21.30	39.32	41.43	72.09	27.91	42.15 #	38.12	11.92	7.81
		EG	26.98	30.13	23.34	19.56	33.90 [c]	11.76	66.06	66.43 [f]	33.57	40.09 ^	28.12 ^	10.65	21.14
		p-value	NS	NS	NS	NS	NS	0.0002	0.0036	NS	NS	NS	NS	NS	0.0271
experience	243(37.02)/ 101(23.09)	PL	9.93 *	12.12	29.51	48.44	23.51	10.30 [c]	66.19	56.88	43.12	12.17	17.88	37.51 $	32.44 $
		EG	19.74	18.06	25.66	36.54	8.57 [c]	25.43	66.00	63.09[f]	36.91	10.90	10.12	42.10 $&	36.88 $&
		p-value	NS	NS	NS	0.0433	0.0013	0.0003	NS	NS	NS	NS	NS	NS	NS
Drug information sheets	152(23.14)/ 132(30.12)	PL	56.41 [h]	9.52	12.09	21.98	39.38	21.30	39.32	41.43	58.57	37.55	29.00	14.14	19.31
		EG	39.59 *	29.31	9.12	21.98	53.34 [c]	33.90	11.76	66.06 [f]	33.94	33.76 #	30.12 #	32.43	3.69
		p-value	0.0047	<0.0001	NS	NS	0.0185	0.0173	<0.0001	<0.0001	<0.0001	NS	NS	0.0002	0.0001
journals	168(25.65)/	PL	42.15 *	38.12	11.92	7.81	43.12 [c]	37.89	18.99	48.12	51.88	56.41 &	9.52	12.09	21.98

	96(21.98)	EG	40.09 [a]	28.12	10.65	21.14	48.76 [c]	29.09	22.15	62.77 [f]	37.23	39.59 ^	29.31	9.12	21.98
		p-value	NS	NS	NS	0.0017	NS	NS	NS	0.0217	0.0217	0.0086	<0.0001	NS	NS
Medical representatives	26(4.03)/ 40(9.12)	PL	42.15 *	38.12	11.92	7.81	71.88 [c]	25.00	3.12	41.35	58.65	37.55 ^	31.96	12.87	17.62
		EG	40.09 [h]	28.12	10.65	21.14	41.65 [d]	22.12	36.23	61.61	38.39	37.34 ^	31.92	2.76	28.98
		p-value	NS	NS	NS	NS	0.0161	NS	0.0018	NS	NS	NS	NS	NS	NS
Internet	512(78.09)/ 360(82.12)	PL	42.01 *	33.23	18.78	5.98	43.12 [c]	37.89	18.99	48.12	51.88	43.09 ^	29.03	7.37	20.51
		EG	34.44 [h]	38.76	10.16	16.64	48.76 [c]	29.09	22.15	62.77 [f]	37.23	52.08 #	26.77	18.09	3.06
		p-value	0.0240	NS	0.0005	<0.0001	NS	0.0070	NS	<0.0001	<0.0001	0.0088	NS	<0.0001	<0.0001
Seminar/conferences	204(31.12)/ 164(37.32)	PL	12.41 *	19.57	38.04	29.98	21.11 [d]	47.58	31.31	58.17	41.83	41.23 $	12.38	27.15	19.24
		EG	15.49*	21.31	29.12	34.08	37.10	25.15	37.75	49.03	50.97	33.76 #	29.12	33.43	3.69
		p-value	NS	NS	0.0490	NS	0.0007	<0.0001	NS	NS	NS	NS	0.0001	NS	<0.0001
Drug promotional literature	40(6.08)/ 53(12.02)	PL	39.10 *	35.43	17.85	7.62	39.76	30.44	29.80	47.43	37.34	29.97 #	34.12 #	30.10 #	5.81
		EG	15.65 *	23.13	16.55	38.27	33.10	37.32	29.58	61.77	38.23	40.09 ^	28.12	10.65	21.14
		p-value	0.0104	NS	NS	0.0007	NS	NS	NS	NS	NS	NS	NS	0.0180	0.0379

PL—Poland; EG—gypt; NS—not statistically significant difference ($p > 0.05$); * statistically significant difference ($p < 0.05$) vs. >50 y.o.a ; [c] significant difference ($p < 0.05$) vs. >20 years; ^ statistically significant difference ($p < 0.05$) vs. private practice/private office; [a] statistically significant difference ($p < 0.05$) vs. 41–50 y.o.a; [d] statistically significant difference ($p < 0.05$) vs. 11–20 years; [f] statistically significant difference ($p < 0.05$) vs. ≥20 average patient/day; [g] statistically significant difference ($p < 0.05$) vs. ≤30 y.o.a.; [h] statistically significant difference ($p < 0.05$) vs. 31–40 years; [j] statistically significant difference ($p < 0.05$) vs. 41–50 y.o.a; # statistically significant difference ($p < 0.05$) vs. other; & statistically significant difference ($p < 0.05$) vs. hospital; $ statistically significant difference ($p < 0.05$) vs. universities
